# Resting-state EEG-based convolutional neural network for the diagnosis of depression and its severity

**DOI:** 10.3389/fphys.2022.956254

**Published:** 2022-10-10

**Authors:** Mengqian Li, Yuan Liu, Yan Liu, Changqin Pu, Ruocheng Yin, Ziqiang Zeng, Libin Deng, Xing Wang

**Affiliations:** ^1^ Department of Psychosomatic Medicine, The First Affiliated Hospital of Nanchang University, Nanchang, China; ^2^ Second Clinical Medical College, Nanchang University, Nanchang, China; ^3^ Queen Mary College, Nanchang University, Nanchang, China; ^4^ Jiangxi Provincial Key Laboratory of Preventive Medicine, School of Public Health, Nanchang University, Nanchang, China; ^5^ School of Public Health, Nanchang University, Nanchang, China; ^6^ School of Life Sciences, Nanchang University, Nanchang, China; ^7^ Clinical Medical Experiment Center, Nanchang University, Nanchang, China

**Keywords:** EEG, convolutional neural network, depression, severity, diagnosis

## Abstract

**Purpose:** The study aimed to assess the value of the resting-state electroencephalogram (EEG)-based convolutional neural network (CNN) method for the diagnosis of depression and its severity in order to better serve depressed patients and at-risk populations.

**Methods:** In this study, we used the resting state EEG-based CNN to identify depression and evaluated its severity. The EEG data were collected from depressed patients and healthy people using the Nihon Kohden EEG-1200 system. Analytical processing of resting-state EEG data was performed using Python and MATLAB software applications. The questionnaire included the Self-Rating Anxiety Scale (SAS), Self-Rating Depression Scale (SDS), Symptom Check-List-90 (SCL-90), and the Eysenck Personality Questionnaire (EPQ).

**Results:** A total of 82 subjects were included in this study, with 41 in the depression group and 41 in the healthy control group. The area under the curve (AUC) of the resting-state EEG-based CNN in depression diagnosis was 0.74 (95%CI: 0.70–0.77) with an accuracy of 66.40%. In the depression group, the SDS, SAS, SCL-90 subscales, and N scores were significantly higher in the major depression group than those in the non-major depression group (*p* < 0.05). The AUC of the model in depression severity was 0.70 (95%CI: 0.65–0.75) with an accuracy of 66.93%. Correlation analysis revealed that major depression AI scores were significantly correlated with SAS scores (r = 0.508, *p* = 0.003) and SDS scores (r = 0.765, *p* < 0.001).

**Conclusion:** Our model can accurately identify the depression-specific EEG signal in terms of depression diagnosis and severity identification. It would eventually provide new strategies for early diagnosis of depression and its severity.

## 1 Introduction

Depression is a common mood disorder that has negative impacts on a patient’s physical and mental health (McCarron et al., 2021; Unursaikhan et al., 2021). The clinical presentation included depressed mood, slowed thinking, and decreased willpower activity. In severe cases, patients might also develop suicidal attempts (Smith, 2014). With the continuous development of human society, the number of people with depression is increasing year by year worldwide (Moreno-Agostino et al., 2020). The World Health Organization (WHO) showed that more than 300 million people worldwide suffer from depression, and about 800,000 of them commit suicide (Levey et al., 2019). Untimely identification of depression may be one of the leading causes of this result. Therefore, early diagnosis of depression is critical.

However, the objectivity and accuracy of depression diagnosis are limited by the current diagnostic criteria for depression. Some facts must be admitted: the diagnostic technique in psychiatry has historically lagged behind other domains (Murray et al., 2021). Fortunately, this challenge is being alleviated by the application of electroencephalogram (EEG) measurement. To date, EEG has been widely used in neuroscience to get insights into brain activity (Latreille et al., 2016; Schönenberg et al., 2017; Wu et al., 2020; Dimitriadis, 2021; Simonato et al., 2021). EEG recordings benefit from shorter test times and lower prices than functional magnetic resonance imaging (fMRI), making them more suitable for diagnosing several types of mental diseases (Čukić et al., 2020). In addition to using traditional EEG images for analysis, the frequency domain features of EEG images have also been shown to be one of the most useful pragmatic markers for diagnosing depression. The frequency domain analysis realizes the conversion of the EEG signal from the time domain to the frequency domain. The frequency domain analysis results in the energy value distribution at each frequency, that is, the power value. For example, Stewart et al. (2010) found that the average alpha power difference measured in the left hemisphere and the right hemisphere in depression patients is larger than that in normal people. Compared with normal people, the left hemisphere activity of depression patients is reduced (expressed as increased alpha power). At the same time, a study suggested that the energy asymmetry of the frontal lobe alpha wave in patients with depression was more obvious to the left, and the severity of symptoms was positively correlated with laterality (Grünewald et al., 2018).

With the rise of computational psychiatry (Geng et al., 2022), EEG-based machine learning (ML) to detect illness phenotypes has attracted growing interest, which provides a theoretical basis and feasibility for disease diagnosis. Since Ahmadlou et al. (2012) initially used ML approaches to detect depression early, much relevant research has been published with promising findings, especially in depression diagnosis (Puthankattil and Joseph, 2012; Hosseinifard et al., 2013; Faust et al., 2014; Bairy et al., 2015). For example, Khodayari-Rostamabad et al. (2010) proposed a diagnostic model trained using EEG data. The model was able to differentiate between subjects with major depressive disorder, chronic schizophrenia, bipolar depression, and healthy subjects by analyzing patients’ EEG data. Meanwhile, Kang et al. (2020) converted the asymmetric features of EEG signals into matrix images, used them as the input of the convolutional neural network, and obtained 98.85% accuracy in depression screening. All of the aforementioned research demonstrates that combining machine learning with EEG signaling can be an effective tool for screening depression patients.

Feature extraction and selection is an essential step in ML, which could improve the model’s performance. Many researchers have proposed various feature extraction and selection methods to improve the performance of resting-state EEG-based ML in differentiating depressed patients from normal controls (Wan et al., 2019; Duan et al., 2020). Despite the advantages mentioned previous to these steps, it has drawbacks, particularly the length of training time needed to obtain reliable classification results. Because of this, more and more researchers have applied deep learning (DL), especially convolutional neural networks (CNNs) (Zhang et al., 2021), to disease diagnosis (Morabito et al., 2017; Ortiz et al., 2021). CNN was a new disease detection model with adaptive learning capability. Its advantage was that, without the need to manually select features, it could shorten the experimental process. Acharya et al. (2018) initially used the CNN to identify the resting-state EEG data on normal and depressed patients with good classification performance, which has attracted significant attention. Many studies have considered that the CNN could be used as a clinically effective computer-aided diagnosis (CAD) system for depression (Kang et al., 2020; Uyulan et al., 2021).

However, the significant limitations to previous studies were two aspects. First, most studies are too strict on resting-state EEG signal preprocessing, which leads to a large number of valuable missing resting-state EEG signals and may overestimate the accuracy of the model. More realistic and high-quality data would help CNN identify the full range of depression in a more clinically meaningful and generalizable way. Second, most studies have only discriminated normal individuals from depression patients without predicting depression severity. It was reported that the severity of depression determined the symptoms, manifestations, and prognosis of the disease (Zimmerman et al., 2018). The clinical potential of deep learning has been undermined by the lack of external validation of models driven by a single dataset and by the increasing use of opaque decision-making frameworks. Therefore, overcoming these challenges is critical to harness the potential of deep learning algorithms to improve patient care and pave the way for interpretable, evidence-based machine learning in the medical imaging community.

It is worth noting that previous studies have been strict with the preprocessing of image information when using EEG modeling (raw EEGs were preprocessed and retained only the image features of depression that had been identified in previous studies). Although this strategy maximizes model accuracy, it also misses the opportunity to discover new depression-specific EEG features. Therefore, we chose to simplify the EEG processing conditions in an attempt to obtain new depression-specific EEG signatures.

Our study ensured realistic and high-quality data by reducing EEG preprocessing and adding EEG screening. In addition, this study would also predict the severity of depression to provide a reliable basis for achieving an accurate diagnosis or clinical decision-making. We chose to simplify the EEG processing conditions in an attempt to obtain new depression-specific EEG features. Using this strategy, we were able to identify new depression-specific EEG signatures in subsequent studies by using techniques such as ‘deconvolutional neural networks’ and to further explore the physiological impact of depression. Our study provides new strategies for the clinical diagnosis of depression.

## 2 Manuscript formatting

### 2.1 Methods

#### 2.1.1 Subjects

A total of 41 depressed patients hospitalized in The First Affiliated Hospital of Nanchang University from September 2020 to April 2021 were selected as the depression group. Meanwhile, 41 healthy people were selected as the healthy control group. Enrollment criteria for the depression group included age 16–65 years, and the patients reached the diagnostic criteria for depression using the International Classification of Diseases, 10th edition (ICD-10). Exclusion criteria included the following: prior diagnosis of somatic disorders, bipolar disorder, schizophrenia, and other psychiatric disorders. Enrollment criteria of the healthy control group included age 16–65 years, and none met the diagnostic criteria for any psychiatric disorder using ICD-10. Exclusion criteria included the following: prior diagnosis of any somatic disorders. Informed verbal consent was obtained for all participants. Moreover, the research was approved by the Research Ethics Board at The First Affiliated Hospital of Nanchang University (approval number: 2022CDYFYYLK(06-030)). All subjects were asked to complete the Self-Rating Depression Scale (SDS) to assess the severity of depression.

#### 2.1.2 Sample for model training/testing

Of the 62 eligible study subjects, there were 30 healthy people (without brain disease), 16 with mild to moderate depression, and 16 with severe depression (the diagnosis of mild, moderate, and moderate depression is performed using the SDS) Each patient’s EEG can be cut into 60 images that meet the requirements. Thus, a total of 1800 images of healthy people, 960 images of patients with mild to moderate depression, and 960 images of patients with severe depression were taken.

Both models use a 10-fold crossover method to divide the image dataset into training and test sets in a ratio of 8:2. In the “Distinguishing Depression Model” (patients with mild to moderate depression and patients with severe depression are divided into a whole), 1440 images of healthy people and 1536 images of patients with depression are included in the training set, and 360 images of healthy people and 384 depression images were included in the test set.

In the “Model for Distinguishing Depression Severity,” 768 images of patients with non-major depression (mild to moderate depression) and 768 images of patients with major depression (severe depression) were included in the training set; 192 images of patients with non-major depression and 192 images of patients with major depression were included in the test set.

#### 2.1.3 Study design

**FIGURE 1:**
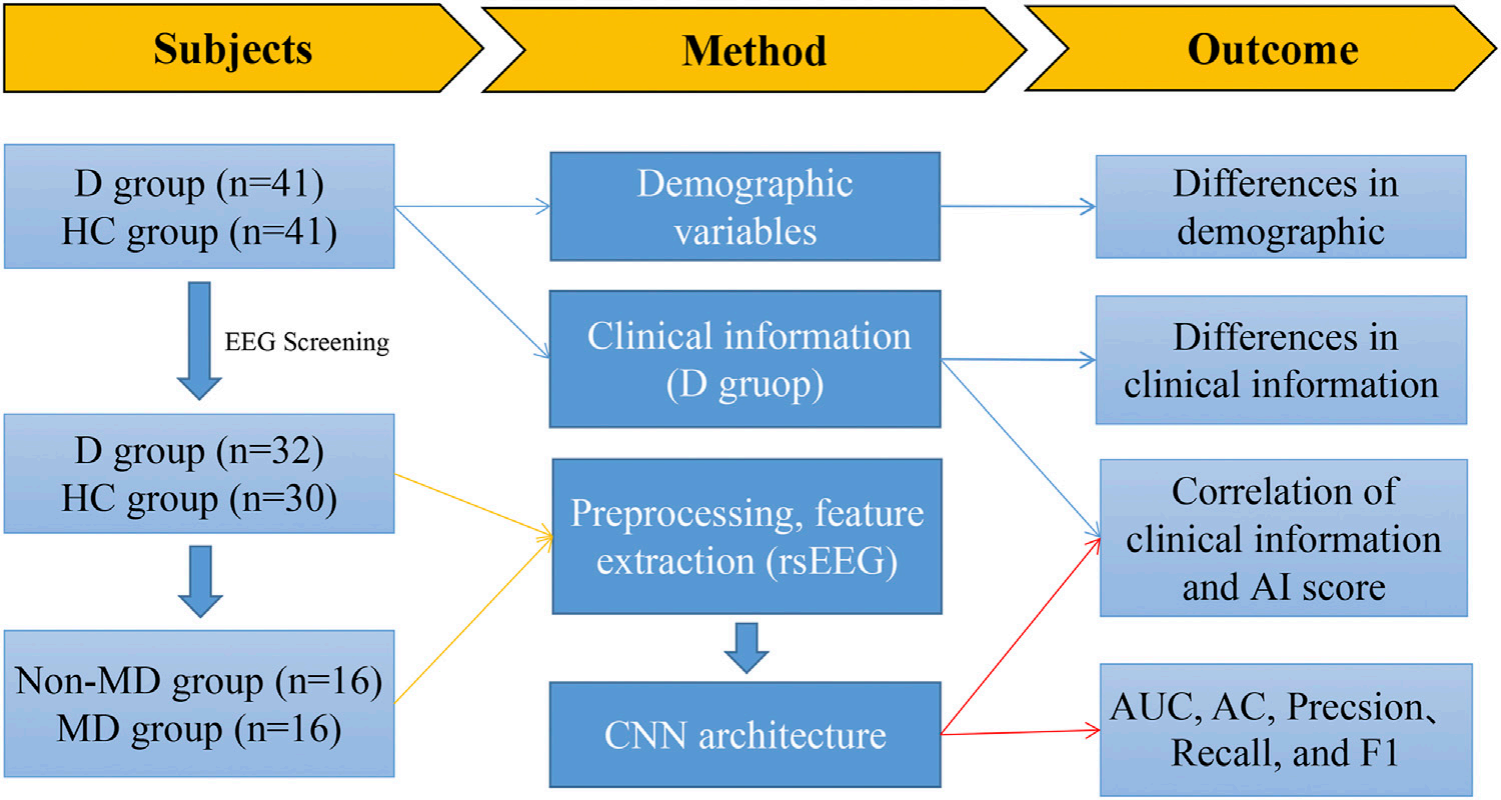
Flowchart illustrating the main content of this study. Note: D group, depression group; HC group, health control group; non-MD group, non-major depression group; MD group, major depression group.

In this study, resting-state EEG signals were collected from the depression group and the healthy control group using the Nihon Kohden EEG-1200 system. A clinical questionnaire survey and disease duration were conducted in the depression group. Gender and age were recorded for all participants. The CNN was used as a classification prediction model for depression. A flowchart of the study is listed as follows (seen in Figure 1).

Due to the characteristics of CNN and the technical limitations to the research group, we cannot use untransformed EEG signals for training (CNN technology uses image data for analysis to obtain different groups of image features and achieve classification). In this study, we selected qualified EEGs of patients who met the requirements and obtained their 600s EEG signals. According to the setting of taking one image in 10 s, we could obtain 19-channel images of a single patient.

##### 2.1.3.1 Measurement

The measurements contained the following four parts: the Self-Rating Anxiety Scale, Self-Rating Depression Scale, Symptom Check-List-90, and the Eysenck Personality Questionnaire.

Self-Rating Anxiety Scale (SAS): The questionnaire is a validated tool for screening anxiety disorders with good reliability and validity (Ding and Yao., 2020; Dunstan et al., 2020). The SAS consisted of 20 items with a total score of 100. A higher score reflects a severer anxiety symptom.

Self-Rating Depression Scale (SDS): This is a widely used measure to screen for depression in clinical settings (Dunstan et al., 2019; Ding and Yao., 2020). The SDS consisted of 20 items. A higher score reflects a severer depression symptom. In this study, we defined a major depression group as having a SDS score≥73. Conversely, the others were defined as non-major depression groups.

Symptom Check-List-90 (SCL-90): This scale is one of the most widely used mental health measures and has high reliability and validity (Derogatis et al., 1973). It consists of 90 items that could be divided into the symptom dimensions of somatization, obsessive-compulsive disorder, interpersonal sensitivity, depression, anxiety, hostility, phobic anxiety, paranoid ideation, and psychoticism. Mental disorder is determined by total score ≥160 points or >2 points for any factor.

Eysenck Personality Questionnaire (EPQ): This is a common self-report personality questionnaire with high validity and reliability. The scale comprised 88 items summarized as extraversion (E), neuroticism (N), psychoticism (P), and lying scales (L) (EPQ, Chinese version) (Gong, 1984). The higher the score, the more likely the patient has the personality traits listed on the scale (Chen et al., 2022).

##### 2.1.3.2 EEG recording

Here, 10 min of resting-state EEG signals were acquired in the eye-closed (EC) conditions according to the 10–20 electrode placement standard. EEG signals were recorded in the frontal (FP1, FP2, F3, F4, F7, F8, and Fz), temporal (T3, T4, T5, and T6), parietal (P3, P4, and Pz), occipital (O1 and O2), and central (C3, C4, and Cz) regions. In addition, we used the bilateral mastoids (A1 and A2) as reference electrodes. EEG signals were collected from 19 channels at a sample rate of 500 Hz. These signals were filtered with a 0.5 Hz–50 Hz bandpass filter and an additional 50-Hz notch filter. The impedance of all electrodes was within a reasonable range.

##### 2.1.3.3 EEG preprocessing and selecting

**FIGURE 2:**
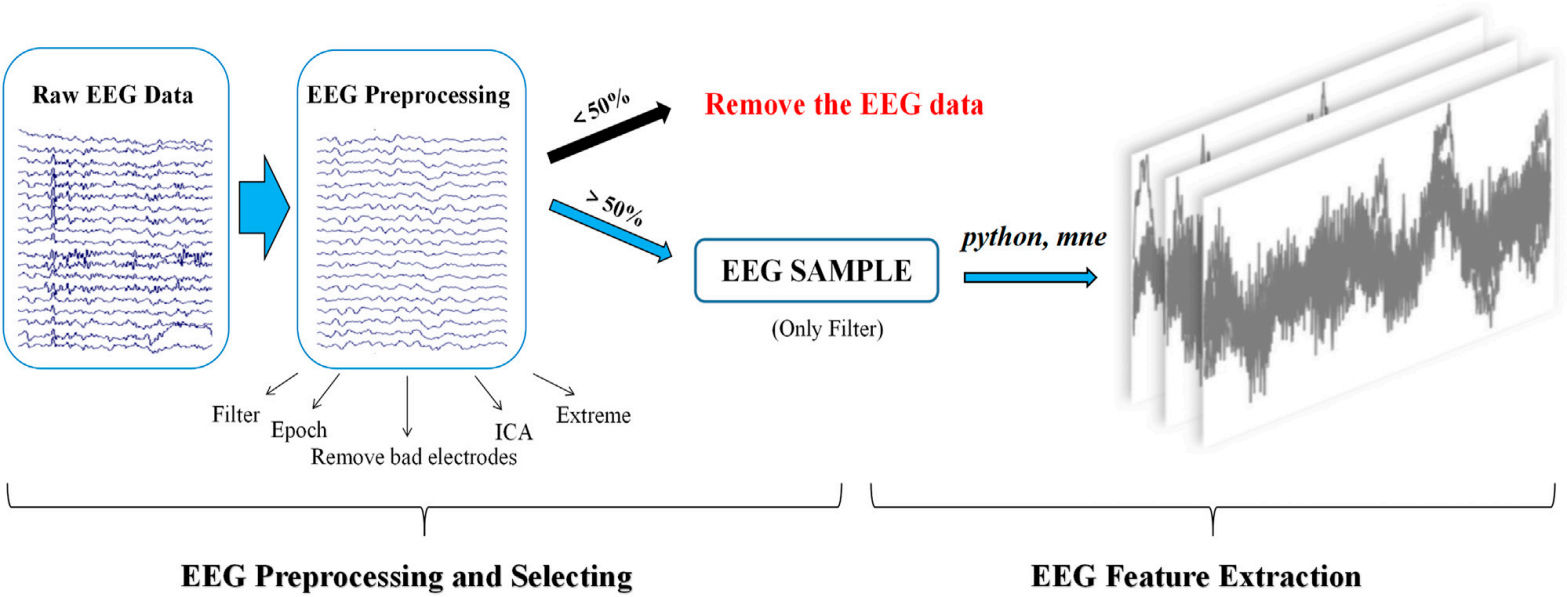
Block diagram for EEG preprocessing, selecting, and feature extraction.

Signal preprocessing of resting-state EEG was performed through the public MATLAB toolbox EEGLAB (Delorme and Makeig, 2004). The steps for selecting resting-state EEG signals with more than 50% effective segments are as follows: first, EEG signals were filtered offline using an FIR bandpass filter (0.5–50 Hz), and then, a notch filter was applied to remove the power frequency interference at 50 Hz. Second, EEG signals were segmented into 2 s long epochs with 300 epochs. Third, bad electrodes were removed with subsequent interpolation. Fourth, independent component analysis (ICA) was applied to identify and remove the eye blink artifacts. Fifth, epochs containing EEG amplitudes that were greater than ±70 uV were rejected automatically. Finally, the effective EEG signals were preprocessed by the aforementioned step 1 and were saved in standard EDF format for future analysis (seen in Figure 2).

##### 2.1.3.4 EEG feature extraction

EEG features were extracted using Python and the MNE toolbox (version 0.23.4) (www.martinos.org/mne). The main steps included the following: 1) the number of electrodes and the amplitude information of each electrode were extracted. 2) The time domain features of the EEG signal were generated by aggregating the amplitude information of all electrodes in the EEG signal. 3) The time domain features were then segmented by a window slide (length of time duration window: 10 s) with a window step of 10 s and no overlap between time duration windows, resulting in a resting-state EEG feature map. For each case of resting-state EEG signals that met the inclusion criteria, 60 images of resting-state EEG features were generated in the PNG format (seen in Figure 2).

##### 2.1.3.5 CNN architecture

**FIGURE 3:**
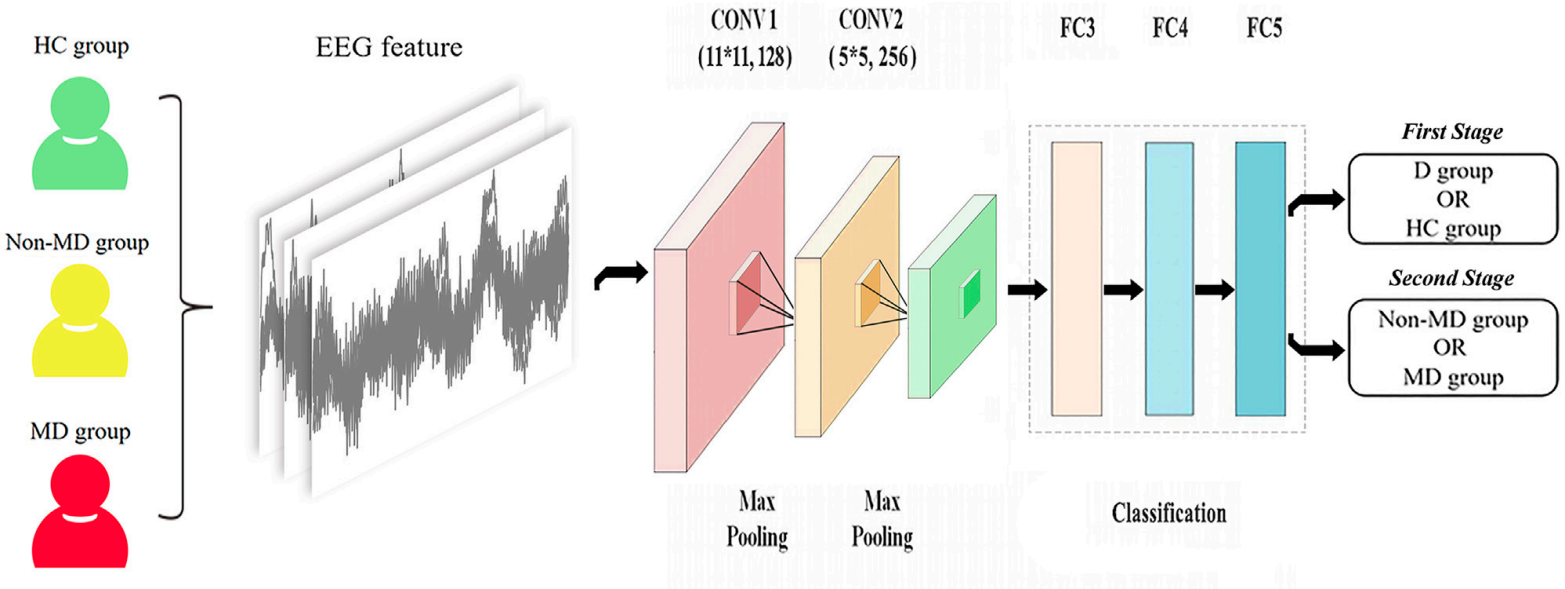
Illustration of the EEG-based CNN for diagnosis of depression and its severity. Note: 1) D group, depression group; HC group, health control group; non-MD group, non-major depression group; MD group, major depression group. 2) CONV1 (11*11, 128): 11*11 represents the size of the convolution kernel, representing height and width, respectively, and 128 represents the number of convolution kernels; CONV2 (5*5, 256): 5*5 represents the size of the convolution kernel, representing height and width, respectively, and 256 represents the number of convolution kernels.

This study adopts a deep learning model using a CNN. The model consisted of three convolutional, two maximum pooling, and three fully connected layers. The convolutional layer extracts the EEG signal features, each with 128, 256 output units. The pooling layer can reduce redundant information. Dropout and L2 regularization were added in the fully connected layers to help prevent overfitting. The network input was a 400 × 400-pixel image. The training set was calculated using stochastic gradient descent. The validation set was used to hyper-parameterize all the networks to finalize the optimal learning rate of 0.001, and the batch size was 32. A total of 32 images were put into the training each time, with a training count of 20, to build the CNN model. The test set was put into the model for testing, and each image was calculated to obtain an AI score from 0 to 1. In fact, each image can obtain the AI score in the model (seen in Figure 3). It illustrates the EEG-based CNN for depression diagnosis and its severity. In our study, the probability of the image being diagnosed with major depression in the depression group was defined as the image’s major depression AI score.

##### 2.1.3.6 Performance evaluation of the CNN

Data were partitioned into three sets (64/16/20) to obtain training, validation, and test sets using the 10-fold cross-validation strategy. The training set was used for model training, while the validation set was used for external validation of the model. The test set was used for evaluating the final performance of the trained model. In addition, we also used the area under the curve (AUC), accuracy (AC), precision, recall, and F1-score (F1) to evaluate the performance of the proposed model. The evaluation metrics are defined as follows:

$$AC=\frac{\left(TP+TN\right)}{\left(TP+FN+TN+FP\right)}$$ (1)

$$\mathrm{Precision}=\frac{TP}{\left(TP+FP\right)}$$ (2)

$$\mathrm{Recall}=\frac{TP}{\left(TP+FN\right)}$$ (3)

$$F1=\frac{\left(2\times TP\right)}{\;\left(2\times TP+FP+FN\right)}$$ (4)

where TP means true positive, TN means true negative, FP means false positive, and FN means false negative.

##### 2.1.3.7 Obtaining the optimal model parameters

In the training set, we use the 10-fold cross-validation method to divide the data into an internal training set and an internal validation set by 8:2. The internal training set participates in the training of the model, and the internal validation set is used to initially evaluate the model’s performance. In this study, we set the number of iterations to 20, and the optimal learning rate is 0.0001. At the same time, during the training process, we can output the accuracy of the internal training set and internal validation set after each iteration. To avoid overfitting, the model with the highest accuracy of the two was chosen, which is regarded as the optimal model (Kiliç et al., 2022).

#### 3.1.4 Statistical analysis

The measurement data conforming to the normal distribution were expressed as means ± standard deviation. Otherwise, the data were expressed as the median (lower and upper quartiles). The independent samples *t*-test or Mann–Whitney *U* test was conducted for the intergroup comparisons accordingly. The count data were expressed as rates and were compared using the chi-squared test. Then, Spearman’s correlation analysis was used to explore the correlation between clinical characteristics and the major depression AI score. *p* < 0.05 was considered to be statistically significant. All data analyses were performed by SPSS 26.0 software.

### 2.2 Results

#### 2.2.1 Demographic characteristics and clinical differences

A total of 82 subjects were included in this study, with 41 in the depression group and 41 in the healthy control group. In the depression group, 31.71% were male, and the median age was 21 years (17–37), with a median disease duration of 1 year (0.38–3). The mean scores of the SAS and SDS were 61.13 ± 2.14 and 69.97 ± 13.39, respectively. The mean scores of the SCL-90 subscales were as follows: somatization (2.47 ± 1.00), obsessive-compulsive disorder (2.98 ± 0.97), interpersonal sensitivity (2.87 ± 1.16), depression (3.21 ± 1.14), anxiety (3.04 ± 1.09), hostility (2.65 ± 1.28), phobic anxiety (2.68 ± 1.15), paranoid ideation (2.50 ± 1.17), and psychoticism (2.60 ± 1.07). The following are the mean scores of the EPQ subscales: E (40.03 ± 14.28), N (62.72 ± 11.69), P (54.37 ± 9.74), and L (41.92 ± 9.53). In the healthy control group, 36.59% were male, and the median age was 28 years (24–47.5). The median age for the healthy control group was higher than that for the depression group (*p* < 0.05). There was no significant difference in gender between the two groups (P > 0.05) (seen in Table 1).

**TABLE 1:** Clinical and demographic characteristics in the depression group and the healthy control group.

	D group (n = 41)	HC group (n = 41)	*χ2/Z*	P
Gender, male (%)	13 (31.71)	15 (36.59)	0.22	0.64
Age (years)	21.00 (17.00, 37.00)	28.00 (24.00, 47.50)	2.49	0.01
Disease duration (years)	1.00 (0.38, 3.00)	—	—	—
SDS (mean, SD)	69.97 ± 13.39	—	—	—
SAS (mean, SD)	61.13 ± 2.14	—	—	—
SCL-90 (mean, SD)				
Somatization	2.47 ± 1.00	—	—	—
Obsessive-compulsive disorder	2.98 ± 0.97	—	—	—
Interpersonal sensitivity	2.87 ± 1.16	—	—	—
Depression	3.21 ± 1.14	—	—	—
Anxiety	3.04 ± 1.09	—	—	—
Hostility	2.65 ± 1.28	—	—	—
Phobic anxiety	2.68 ± 1.15	—	—	—
Paranoid ideation	2.50 ± 1.17	—	—	—
Psychoticism	2.60 ± 1.07	—	—	—
EPQ (mean, SD)				
E	40.03 ± 14.28	—	—	—
N	62.72 ± 11.69	—	—	—
P	54.37 ± 9.74	—	—	—
L	41.92 ± 9.53	—	—	—

D group, depression group; HC, group, health control group; E, extraversion; N, neuroticism; P, psychoticism; L, lying scales.

The depression group consists of 22 patients with non-major depression and 19 patients with major depression. The SDS, SAS, SCL-90 subscales, and N score were significantly higher in the major depression group than those in the non-major depression group (*p* < 0.05), whereas for E, P, and L, no difference existed among groups. In addition, the median age for the non-major depression group was higher than that for the major depression group (*p* < 0.05). There was no significant difference in gender between the two groups (P > 0.05) (seen in Table 2).

**TABLE 2:** Clinical and demographic characteristics in the non-major depression group and the major depression group.

	Non-MD group (n = 22)	MD group (n = 19)	*χ2/Z/t*	P
Gender, male (%)	7 (31.82)	6 (31.58)	0	0.99
Age (years)	25.00 (20.00, 42.00)	18.00 (16.00, 26.00)	−2.51	0.01
Disease duration (years)	1.00 (0.25, 5.25)	1.00 (0.50, 2.00)	−0.07	0.95
SDS (mean, SD)	63.75 (56.25, 69.06)	78.75 (76.25, 85.00)	−5.47	<0.001
SAS (mean, SD)	53.47 ± 10.92	70.00 ± 11.16	−4.79	<0.001
SCL-90 (mean, SD)				
Somatization	2.07 ± 0.87	2.95 ± 0.95	−3.10	0.004
Obsessive-compulsive disorder	2.53 ± 0.92	3.51 ± 0.73	−3.73	0.001
Interpersonal sensitivity	2.28 ± 1.01	3.56 ± 0.94	−4.17	<0.001
Depression	2.53 ± 0.98	4.01 ± 0.72	−5.45	<0.001
Anxiety	2.50 ± 0.98	3.67 ± 0.87	−4.04	<0.001
Hostility	2.27 ± 1.39	3.10 ± 0.98	−2.17	0.04
Phobic anxiety	2.18 ± 1.05	3.26 ± 1.00	−3.39	0.002
Paranoid ideation	1.91 ± 0.90	3.18 ± 1.09	−4.10	<0.001
Psychoticism	2.04 ± 0.82	3.26 ± 0.96	−4.41	<0.001
EPQ (mean, SD)				
E	43.30 ± 15.89	36.25 ± 11.42	1.61	0.12
N	58.76 ± 12.76	67.30 ± 8.50	−2.48	0.02
P	53.14 ± 10.76	55.78 ± 8.48	−0.86	0.39
L	41.80 ± 11.44	42.05 ± 7.00	−0.08	0.94

Note: non-MD, group, non-major depression group; MD, group, major depression group; E, extraversion; N, neuroticism; P, psychoticism; L, lying scales.

#### 2.2.2 Resting-state EEG screening

In the depression group, the resting-state EEG data met the inclusion criteria in 32 cases, with an inclusion rate of 78.05%, and the number of valid segments per EEG data was 231.33 ± 47.74. In the healthy control group, their resting-state EEG data met the inclusion criteria in 30 cases, with an inclusion rate of 73.17% and a valid number of segments per EEG data of 225.61 ± 41.29.

In the non-major depression group, the resting-state EEG data met the inclusion criteria in 16 cases, with an inclusion rate of 72.73% and a valid number of segments per EEG data (238.69 ± 45.26). In the major depression group, the resting-state EEG data met the inclusion criteria in 16 cases, with an inclusion rate of 84.21% and a valid number of segments per EEG data (223.06 ± 45.81).

#### 2.2.3 CNN performance

**FIGURE 4:**
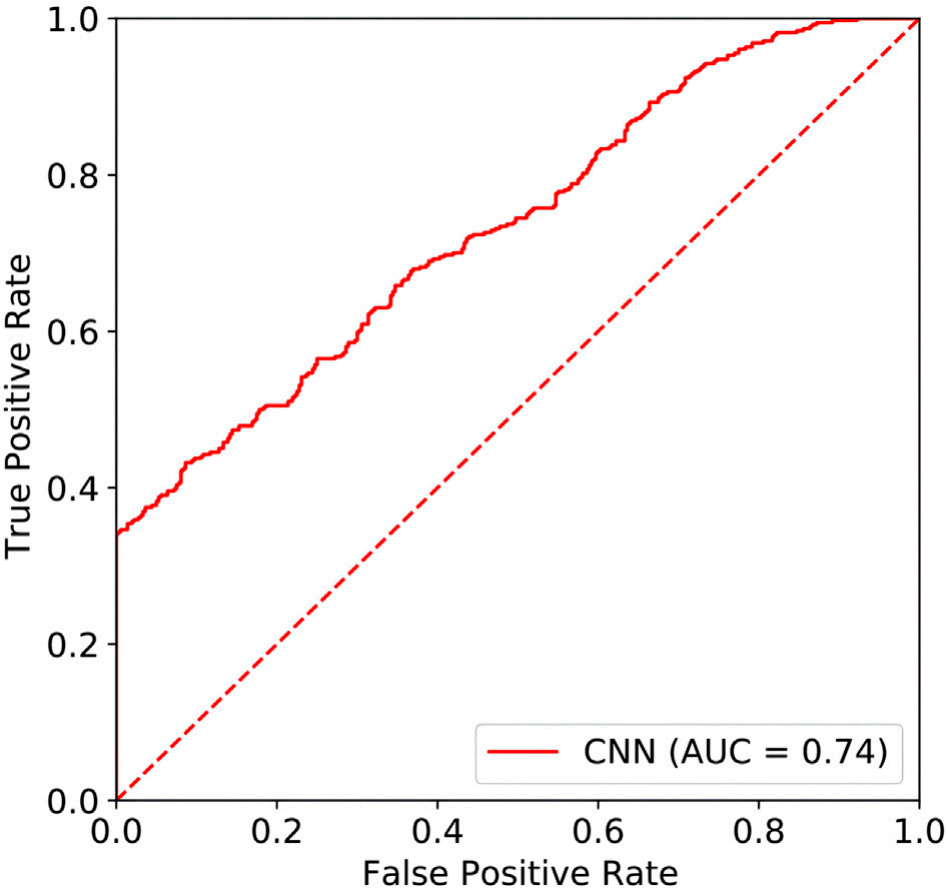
ROC of the EEG-based CNN in depression diagnosis.

**FIGURE 5:**
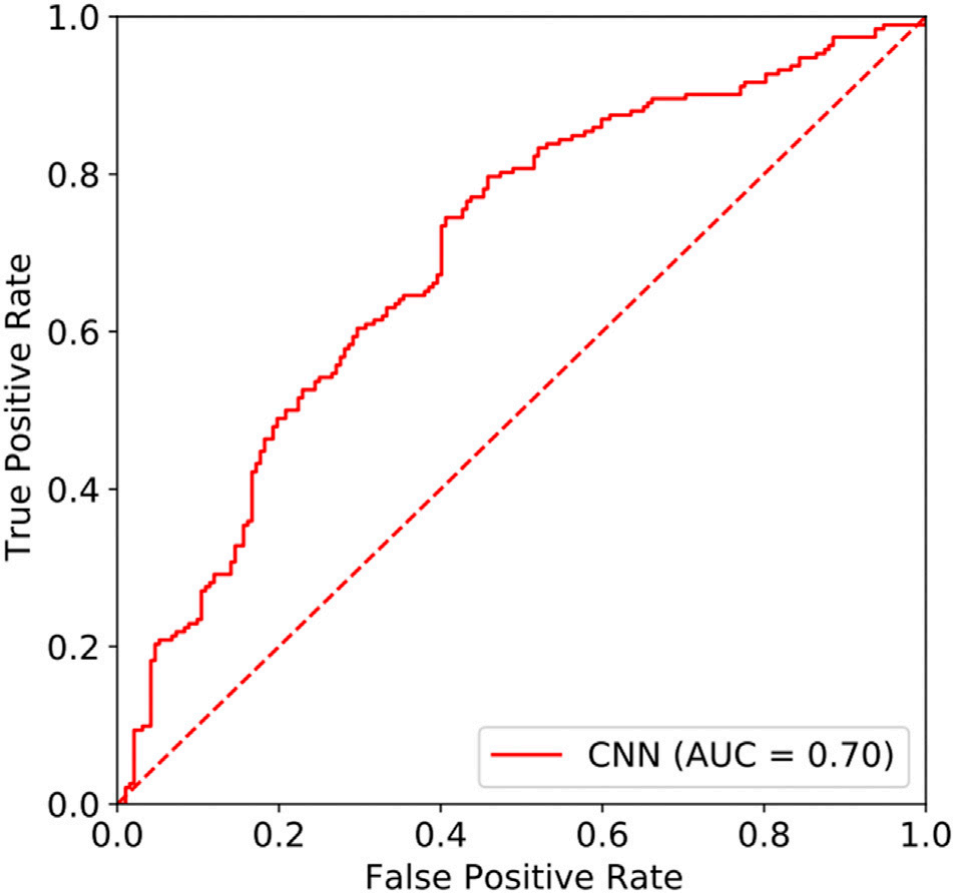
ROC of the EEG-based CNN in depression severity.

In this study, the area under the curve (AUC) of the resting-state EEG-based CNN in depression diagnosis was 0.74 (95%CI: 0.70–0.77) (seen in Figure 4), with an accuracy of 66.40%, precision of 83.84%, recall of 43.23%, and F1 score of 57.04%. In addition, the AUC of the resting-state EEG-based CNN in depression severity was 0.70 (95%CI: 0.65–0.75) (seen in Figure 5), with an accuracy of 66.93%, precision of 63.49%, recall of 79.69%, and F1 score of 70.67% (seen in Table 3).

**TABLE 3:** Performance of the CNN model in depression diagnosis and its severity.

	AUC	Accuracy (%)	Precision (%)	Recall (%)	F1 score (%)
Depression diagnosis	0.74 (95%CI: 0.70–0.77)	66.40	83.84	43.23	57.04
Depression severity	0.70 (95%CI: 0.65–0.75)	66.93	63.49	79.69	70.67

Note: 95% CI: 95% confidence interval.

#### 2.2.4 Correlation of clinical characteristics and major depression AI score

**FIGURE 6:**
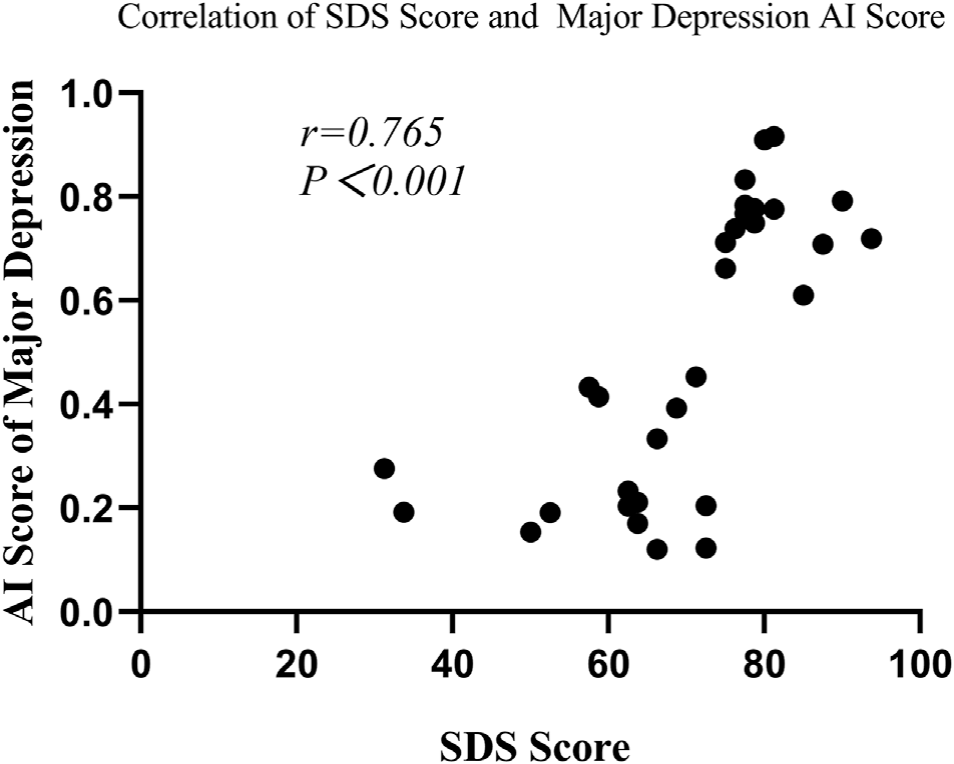
Correlation of the SDS score with the major depression AI score.

**FIGURE 7:**
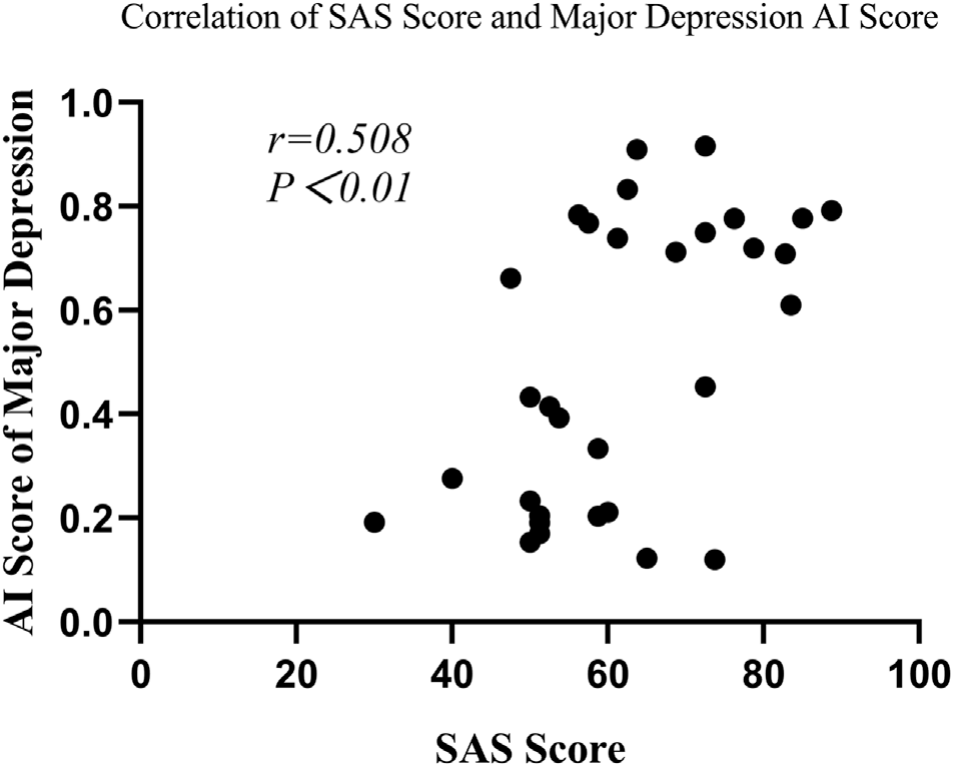
Correlation of the SAS score with the major depression AI score.

In the depression group, Spearman’s correlation analysis revealed that major depression AI scores were significantly correlated with SAS scores (r = 0.508, *p* = 0.003) and SDS scores (r = 0.765, *p* < 0.001) (seen in Figure 6 and Figure7), but the scores were not remarkably correlated with EPQ subscale scores (P, r = 0.011, *p* = 0.953; E, r = -0.305, *p* = 0.090; N, r = 0.322, *p* = 0.072; L, r = 0.208, *p* = 0.253).

The analysis was also performed between major depression AI scores and SCL-90 subscale scores. It showed that major depression AI scores were significantly correlated with somatization (r = 0.492, *p* = 0.004), obsessive-compulsive disorder (r = 0.546, *p* = 0.001), interpersonal sensitivity (r = 0.530, *p* = 0.002), depression (r = 0.653, *p* < 0.001), anxiety (r = 0.506, *p* = 0.003), hostility (r = 0.496, *p* = 0.004), phobic anxiety (r = 0.485, *p* = 0.005), paranoid ideation (r = 0.522, *p* = 0.002), and psychoticism (r = 0.531, *p* = 0.002).

### 2.3 Discussion

To the best of our knowledge, there was no previous study that added the EEG screening process in their research. The step was critical considering the vulnerability of the EEG signal (Müller-Putz, 2020). In this study, the AUC of the resting-state EEG-based CNN for differentiating between depression patients and healthy people was 0.74, with an accuracy of 66.40%. The accuracy was within a close approximation to the findings of the previous study (Cai et al., 2018). However, our model got lower AUC and accuracy in depression diagnosis compared to the results of most previous studies (Acharya et al., 2018; Kang et al., 2020; Uyulan et al., 2021). This might be associated with the following reasons: first, some EEG artifacts might be retained, owing to the fewer preprocessing steps (Jiang et al., 2019). The residual EEG artifacts tend to have a great impact on the CNN performance. Second, the amount of data included in this study was relatively small compared to the previous studies. This also might have influenced the performance of the classification model (Li et al., 2011). Despite that, more steps of EEG preprocessing and feature selection were disadvantageous for assisting clinicians in rapid decision-making. As a result, it suggested that CNN’s accuracy in clinical depression diagnosis might have been exaggerated in previous studies.

Furthermore, our study also evaluated depression severity with the model. Similar to the results of previous studies, SDS, SAS, SCL-90 subscales, and N score were significantly higher in the major depression group than those in the non-major depression group. Because people who have co-morbidity with anxiety are more likely to suffer from depression, the enhanced level of anxiety might be related to that of negative emotions (Goldberg and Fawcett, 2012; Choi et al., 2020). In addition, the impairment of cognitive function and strained interpersonal relationships was identified to be the most strongly associated with depression severity (Douglas et al., 2018). Furthermore, this study found that major depression patients had a higher risk of psychotic symptoms (Dubovsky et al., 2021). Major depression with psychotic symptoms tended to have a higher risk of comorbidity and suicide (Gaudiano et al., 2009; Dold et al., 2019), which had a greater impact on the quality of life of patients (Wang et al., 2021). Meanwhile, worse results were anticipated with the treatment of pharmacotherapy and psychotherapy in major depression with psychotic symptoms (Craig et al., 2007; Dold et al., 2019). Therefore, it was particularly important to accurately identify major depression. In this study, the AUC of the resting-state EEG-based CNN for differentiating between non-major depression patients and major depression patients was 0.70 with an accuracy of 66.93%. Although different modeling methods and data processing strategies were used, the AUC and accuracy of our model were close to those of previous studies (Dibeklioglu et al., 2018; Mahato et al., 2020; Kwon and Kim, 2021). It might suggest that the model has better performance and stability in depression grading of severity. Furthermore, we found that major depression AI scores were positively correlated with depression symptoms, which further clarified the aforementioned result.

In this study, major depression AI scores were also positively correlated with anxiety symptoms, somatization, obsessive-compulsive disorder, interpersonal sensitivity, hostility, phobic anxiety, paranoid ideation, and psychoticism. These results further showed that the aforementioned symptoms were remarkably associated with major depression and indicated that depressed patients with the symptoms were prone to diagnosis with major depression in our model. Meanwhile, it somewhat indicated that the model could be generalized to identify other psychiatric disorders associated with the aforementioned symptoms in the future. In particular, schizophrenia (Shoeibi et al., 2021), bipolar disorder (Li et al., 2021), anxiety disorders (Xing et al., 2019), and obsessive-compulsive disorder (Cohn et al., 2018) should be considered. Unfortunately, we did not find a significant correlation between major depression AI scores and EPQ subscale scores, which were approximately consistent with the clinical results except for neuroticism. It might indicate that personality traits have a limited contribution to identifying depression severity. Meanwhile, it is worth noting that the aforementioned conclusions still should be considered with caution, owing to the restrictions of our results. More analysis will be performed in future research.

In addition, there were still some limitations to this study: first, the lack of questionnaire information on the healthy people and the lack of clinical information on the patients, such as treatment with antidepressants. Second, the groups did not match in age. Third, using a single EEG signal as a data-driven ML model for depression diagnosis lacks clinical value and accuracy compared to existing ML models that include multimodal data. Fourth, we did not build a “diagnostic model of depression” by grouping mild, moderate, and severe image data. Further research can group the three types of data and models separately to obtain a more accurate diagnosis model of depression.

Therefore, further studies with more rigorous experimental design and clinical information are expected. In particular, regional cooperation and multi-center research studies should be encouraged (Geng et al., 2022). The most recent approaches to the diagnosis of depression have focused primarily on graph theory in neuropsychiatry (Aydin et al., 2022; Kilic et al., 2022). Using GT-based network analysis, researchers can estimate global connectivity measures from multichannel EEG recordings. We will pay more attention to network measurement based on graph theory in future research directions. Meanwhile, we would continue to integrate multi-model data such as EEG, fMRI, and DNA methylation data to create more accurate artificial prediction models, eventually providing new strategies for early depression diagnosis and its severity.

## 3 Conclusion

In this study, our model can accurately identify the depression-specific EEG signal, both in terms of depression diagnosis and its severity identification. Based on this, we conclude that the model could be a useful aid for depression diagnosis and its severity. It would eventually provide new strategies for early depression diagnosis and its severity.

## Data Availability

The raw data supporting the conclusions of this article will be made available by the authors, without undue reservation.
